# International prospective observational cohort study of Zika in infants and pregnancy (ZIP study): study protocol

**DOI:** 10.1186/s12884-019-2430-4

**Published:** 2019-08-07

**Authors:** Jill F. Lebov, Juan F. Arias, Angel Balmaseda, William Britt, José F. Cordero, Luiz Augusto Galvão, Ana Lucía Garces, K. Michael Hambidge, Eva Harris, Albert Ko, Nancy Krebs, Ernesto T. A. Marques, Alexander M. Martinez, Elizabeth McClure, Democrito B. Miranda-Filho, Maria Elisabeth Lopes Moreira, Marisa M. Mussi-Pinhata, Theresa J. Ochoa, Jorge E. Osorio, Deolinda M. F. Scalabrin, Stacey Schultz-Cherry, George R. Seage, Kristen Stolka, César Augusto Ugarte-Gil, Carmen Milagros Velez Vega, Michael Welton, Ricardo Ximenes, Carmen Zorrilla

**Affiliations:** 1 Social, Statistical and Environmental Sciences, RTI International, Durham, NC USA; 2 Department of Infectious Diseases, St Jude Children’s Research Hospital, Memphis, TN 38105 USA; 3 Centro Nacional de Diagnostico y Referencia, Complejo Nacional de Salud, Managua, Nicaragua; 4 Department of Pediatrics, University of Alabama at Birmingham, Birmingham, AL USA; 5 Department of Epidemiology and Biostatistics, College of Public Health, University of Georgia, Athens, GA USA; 6 Center for Global Health – CRIS, FIOCRUZ, Rio de Janeiro, Brazil; 7 Fundación para la Alimentación y Nutrición de Centro América y Panamá (INCAP), Guatemala City, Guatemala; 8 Section of Nutrition, Pediatrics, University of Colorado, Aurora, CO USA; 9 Division of Infectious Diseases and Vaccinology, School of Public Health, University of California, Berkeley, CA USA; 10 Department of Epidemiology of Microbial Diseases, Yale School of Public Health, New Haven, CT USA; 11 Instituto Gonçalo Moniz, Fundação Oswaldo Cruz/MS, Salvador, Brazil; 12 School of Public Health, University of Pittsburgh, Pittsburgh, PA USA; 13 Instituto Aggeu Magalhães, Department of Virology and Experimental Therapeutics, FIOCRUZ, Pernambuco, Brazil; 14 Director of Research Institute at Imbanaco Medical Center, Cali, Colombia; 15 Programa de Pós-Graduação em Ciências da Saúde (PPGCS) da Universidade de Pernambuco, Microcephaly Epidemic Research Group, Recife, Brazil; 16 Instituto Fernandes Figueira – FIOCRUZ, Rio de Janeiro, Brazil; 17 Ribeirão Preto Medical School, Ribeirão Preto, Brazil; 18 Instituto de Medicina Tropical Alexander von Humboldt and Facultad de Medicina, Universidad Peruana Cayetano Heredia, Lima, Peru; 19 Department of Pathobiological Sciences, University of Wisconsin, Madison, WI USA; 20 Department of Epidemiology, Harvard Chan School of Public Health, Boston, MA USA; 21 University of Puerto Rico, San Juan, Puerto Rico; 22 Departamento de Medicina Tropical da Universidade Federal de Pernambuco, Microcephaly Epidemic Research Group, Recife, Brazil; 23 Maternal-Infant Studies Center (CEMI), San Juan, Puerto Rico

**Keywords:** Zika virus, Pregnancy, Latin America, Microcephaly

## Abstract

**Background:**

Until recently, Zika virus (ZIKV) infections were considered mild and self-limiting. Since 2015, they have been associated with an increase in microcephaly and other birth defects in newborns. While this association has been observed in case reports and epidemiological studies, the nature and extent of the relationship between ZIKV and adverse pregnancy and pediatric health outcomes is not well understood. With the unique opportunity to prospectively explore the full spectrum of issues related to ZIKV exposure during pregnancy, we undertook a multi-country, prospective cohort study to evaluate the association between ZIKV and pregnancy, neonatal, and infant outcomes.

**Methods:**

At research sites in ZIKV endemic regions of Brazil (4 sites), Colombia, Guatemala, Nicaragua, Puerto Rico (2 sites), and Peru, up to 10,000 pregnant women will be recruited and consented in the first and early second trimesters of pregnancy and then followed through delivery up to 6 weeks post-partum; their infants will be followed until at least 1 year of age. Pregnant women with symptomatic ZIKV infection confirmed by presence of ZIKV RNA and/or IgM for ZIKV will also be enrolled, regardless of gestational age. Participants will be tested monthly for ZIKV infection; additional demographic, physical, laboratory and environmental data will be collected to assess the potential interaction of these variables with ZIKV infection. Delivery outcomes and detailed infant assessments, including physical and neurological outcomes, will be obtained.

**Discussion:**

With the emergence of ZIKV in the Americas and its association with adverse pregnancy outcomes in this region, a much better understanding of the spectrum of clinical outcomes associated with exposure to ZIKV during pregnancy is needed. This cohort study will provide information about maternal, fetal, and infant outcomes related to ZIKV infection, including congenital ZIKV syndrome, and manifestations that are not detectable at birth but may appear during the first year of life. In addition, the flexibility of the study design has provided an opportunity to modify study parameters in real time to provide rigorous research data to answer the most critical questions about the impact of congenital ZIKV exposure.

**Trial registration:**

NCT02856984. Registered August 5, 2016. Retrospectively registered.

## Background

Until recently, Zika virus (ZIKV) infections were considered mild and self-limiting; however, since 2015, they have been associated with an increase in microcephaly and other birth defects in newborns [1–6].

ZIKV, first identified in 1947 in Uganda, is a single-stranded RNA virus of the RNA virus family *Flaviviridae* that includes other human pathogenic viruses such as dengue virus (DENV) and West Nile virus (WNV) [7]. Between the first isolation of ZIKV in monkeys in 1947 until 2007, reports of human cases had been sporadic [1, 2]. Although the virus was isolated widely in Africa and Southeast Asia, infections were largely asymptomatic, and no epidemics had been observed [1, 2]. Then, in 2007, an outbreak of the Asian lineage of ZIKV occurred in Federated States of Micronesia. In French Polynesia, more than 30,000 suspected ZIKV cases were reported within a 6-month period in 2013 [8].

There were no reported cases of ZIKV in the Western Hemisphere prior to 2014. The first cases of ZIKV in the Americas were reported in northeast Brazil in 2015 [9–11]. Because of the lack of immunity in the population, and the abundance of *Aedes aegypti* mosquitos in Brazil, an autochthonous transmission was established with ensuing widespread dissemination of ZIKV [9]. Although initially concentrated in northeastern Brazil, the virus has since spread throughout South and Central America, the Caribbean, and Puerto Rico [10].

With the introduction of ZIKV, Brazil witnessed an epidemic of microcephaly cases among newborns. In 2015, the Brazilian Ministry of Health (MoH) observed an increased incidence of microcephaly in newborns in the northeastern part of the country in the states of Pernambuco, Paraíba and Rio Grande do Norte, in association with clusters of ZIKV-like illness in pregnant women [12]. To evaluate the association between microcephaly and congenital Zika virus infection, investigators in Pernambuco carried out a case-control study among 91 microcephaly cases (defined as head circumference of 2 or more standard deviations below the mean, per Intergrowth-21 standards) and 173 controls. The results of this study showed a strong association between microcephaly and laboratory confirmed Zika virus infection (Odds Ratio: 73.1; 95% CI 13·0–∞), after adjusting for confounders, and none of the other risk factors studied, including use of the larvicide, pyriproxyfen, nor vaccine administration during pregnancy, was associated with microcephaly in multivariable analyses [13].

Microcephaly had not been associated with ZIKV infection prior to the outbreak in the Americas, but this association had not been examined systematically during previous outbreaks. Using evidence available as of early 2016, Rasmussen et al. used Shepherd’s criteria and Bradford Hill criteria to infer a causal relationship between prenatal ZIKV infection and birth defects [14]. In a recent retrospective review, Besnard et al. described 10 cases of congenital cerebral malformations in fetuses and newborns from the 2013–2014 ZIKV outbreak in French Polynesia [15]. Congenital abnormalities associated with ZIKV infection have since been reported in Colombia and the United States, and in other countries in South America [11, 12, 16–18]. Of 972 pregnant women enrolled in the US Zika Pregnancy Registry (USZPR) with laboratory evidence of a possible ZIKV infection, 5% had a fetus or infant with evidence of a ZIKV-related birth defect [19]. A higher than expected prevalence of microcephaly was observed in Cali and Bogota, Colombia, during the peak ZIKV epidemic period [1].

“Congenital Zika syndrome” (CZS) reflects a pattern of birth defects observed in affected infants [20, 21]. The full spectrum of fetal and infant outcomes associated with ZIKV infection during pregnancy is currently unknown, but recent studies have observed a range of outcomes, including brain abnormalities and neural tube defects, ocular abnormalities, and symptoms related to central nervous system dysfunction (e.g. arthrogryposis, deafness, and microcephaly resulting from fetal brain disruption sequence) [22, 23]. In a study of 46 presumed CZS cases age 1 to 8 months in Maranhão, Brazil, the most commonly observed central nervous system symptoms were irritability (85%), pyramidal/extrapyramidal syndrome (56%), epileptic seizures (50%) and dysphagia (15%) [2].

Infants with ZIKV-associated microcephaly may have comorbid ocular and hearing abnormalities. In a study conducted in Salvador, Brazil, 10 out of 29 infants with presumed ZIKV-associated microcephaly had ocular abnormalities [24]. In a study of the infants of 112 ZIKV-infected mothers, 24 had eye abnormalities, and the presence of abnormalities was associated with microcephaly (odds ratio: 19.1; 95% CI, 6.0–61.0) [25]. Among 69 infants aged 0–10 months with microcephaly and laboratory evidence of ZIKV infection, 4 had sensorineural hearing loss [26]. In imaging studies, brain abnormalities such as intracranial calcifications, cerebral atrophy, corpus collosum abnormalities, cortical malformations, ventriculomegaly, and hydrocephaly, have been observed among fetuses and neonates with presumed in utero exposure to ZIKV [16, 27–29].

To date, most studies have included only symptomatic women. A report of 88 pregnant women with a rash in Rio de Janeiro revealed that 82% tested positive for ZIKV in blood or urine. In addition to rash, women had arthralgias, conjunctival injection, and headache [30]. Twenty-nine percent of ZIKV-positive women had abnormal ultrasounds with corresponding adverse outcome of pregnancies (fetal demise or newborns with confirmed pathology). A cohort study of pregnant women with rash in Rio de Janeiro, Brazil, reported the findings of 134 ZIKV-positive women and 73 ZIKV-negative women [11]. Among 125 ZIKV-positive women for whom outcomes of pregnancy were available, adverse pregnancy outcomes were observed in 46.4%, and among 117 live-born infants, 42% had abnormalities on clinical examination, imaging, or both; 3.4% had microcephaly. Thus, self-limited symptomatic infections during pregnancy were associated with severe outcomes in the fetus. Information is limited about the incidence of fetal abnormalities among women who have asymptomatic ZIKV infection during their pregnancy. In the USZPR study, birth defects were reported in similar proportions of fetuses/infants whose mothers did and did not report symptoms [19]. Other studies have not been able to capture data on asymptomatic women.

Increasing evidence points to ZIKV as the agent responsible for a variety of birth defects in newborns of mothers who become infected during pregnancy [16, 31, 32]. However, the strength of the causal relationship of ZIKV infections in pregnancy with adverse outcomes has yet to be determined. Most studies to date have followed women known to be infected or infants who were born with serious birth defects. Previous studies have not been able to determine differences in risk of ZIKV-associated outcomes by trimester of infection, the rate of vertical transmission and factors that affect this rate, and the full spectrum of infant health outcomes associated with congenital ZIKV exposure.

To address these gaps, we have undertaken a prospective cohort study among pregnant women in areas in Latin America with local ZIKV transmission, including Brazil, Colombia, Guatemala, Nicaragua, Puerto Rico, and Peru. The overall objective of this multisite, multi-country Zika in Infants and Pregnancy (ZIP) study is to assess the strength of the association of ZIKV infection during pregnancy with adverse maternal, fetal, and infant outcomes and the risk of vertical transmission overall, by gestational age, and by presence of symptoms during pregnancy.

## Methods

### Study design

This prospective, international, multisite cohort Zika in Infants and Pregnancy (ZIP) study will recruit up to 10,000 pregnant women from ZIKV-endemic regions and follow them longitudinally throughout pregnancy, delivery, and 6 weeks postpartum. Putative cases of ZIKV infection among pregnant women will be identified by monitoring for symptoms of ZIKV-like illness and performing serial laboratory sampling for diagnosis of seroconversion and viral shedding. Infants born to women enrolled in the study will be followed through 1 year of age and evaluated for neurological, audiological, ophthalmological, and other clinical outcomes.

### Study population

ZIP will recruit pregnant women within the first or early second trimester (up to and including 17 weeks and 6 days), age 15 years and older (with assent and consent as required per local country regulations). Additionally, any pregnant women, regardless of gestational age, with acute Zika-like symptoms confirmed by serology and/or RT-PCR are eligible. Recruitment will occur at hospitals and health clinics across Brazil, Colombia, Guatemala, Nicaragua, Puerto Rico, and Peru. At each of these sites, participants may be referred to this study from surrounding clinical centers. Any pregnant women enrolled in other clinical research (including other Zika studies) requiring blood sampling that, in combination with ZIP requirements, exceeds 50 cc every 8 weeks will not be included in ZIP. Women who indicate at screening that they cannot adhere to the proposed visit schedule are also excluded from the study. Infants are eligible for enrollment if they were born to women enrolled in ZIP and have parental consent.

### Study objectives

#### Primary objectives

- To compare the occurrence of congenital malformations and adverse outcomes (including microcephaly, fetal demise, neonatal death, CNS malformations, hydrops, and ocular abnormalities) in fetuses/infants of women who become infected with ZIKV during pregnancy compared to women who do not.
- To compare the occurrence of congenital malformations and adverse outcomes in fetuses/infants of women who become symptomatic with ZIKV infection during pregnancy versus those who do not.

#### Secondary objectives

- To determine the long-term clinical sequelae among infected infants.
- To determine the rate of ZIKV seroconversion among pregnant women from ZIKV-endemic regions.
- To compare the severity of maternal and infant outcomes by gestational age, stratified by symptomatic and asymptomatic ZIKV infection.
- To compare occurrence of adverse maternal and fetal outcomes (including preeclampsia, preterm birth, intrauterine growth restriction (IUGR), and oligohydramnios/polyhydramnios) between women who become infected with ZIKV infection during pregnancy and women who do not.
- To compare occurrence of adverse maternal and fetal outcomes (including preeclampsia, preterm birth, IUGR, and oligohydramnios/polyhydramnios) between women with symptomatic vs. asymptomatic ZIKV infection.
- To determine if coinfections and cofactors (including social and environmental factors) contribute to the presence and severity of CNS malformations and disease in the offspring.
- To determine the duration of ZIKV persistence and shedding during pregnancy in blood, urine, genital secretions, and saliva.
- To determine whether mothers with ZIKV infection prior to or after delivery shed virus in breast milk and to determine the incidence of postpartum infant ZIKV infection acquisition.

### Study procedures

At study entry, women will be interviewed or have data abstracted about their medical, vaccination, and pregnancy history. At each monthly visit during pregnancy, at delivery, and 6-weeks (+/− 2 weeks) post-partum, women will have a physical exam or have data abstracted from their medical chart and be administered a health questionnaire on general and pregnancy health indicators, exposure to environmental hazards, and evidence of prior ZIKV symptoms. All women will have samples (blood, urine, saliva, and vaginal fluids) collected for assessment of ZIKV and other potential infections (i.e., Toxoplasmosis, Rubella, Cytomegalovirus (CMV), and Herpes Simplex Virus (HSV) (TORCH) and syphilis), and environmental exposures. Additional TORCH testing may be conducted per the local standard of care. Detailed delivery information will be obtained about delivery circumstances and outcomes.

If women note that they will be getting an amniocentesis for clinical care, the study team will coordinate with the participant and her obstetrician to obtain a sample of fluid for testing for ZIKV infection. Breastmilk will be collected, if possible, at the delivery and 6-weeks post-partum visit. If the delivery is a live birth, cord blood, placental tissue, and amniotic fluid will be collected during the delivery visit, if possible. Fetal tissues will be collected, if possible, in the event of a fetal loss.

In between monthly visits, women will provide a urine sample, which is collected at home and brought to the next monthly visit or collected at a biweekly visit in the clinic. This sample will be retrospectively tested for ZIKV if she has a serologically positive test for ZIKV at a monthly visit. Women will also be asked to keep track of potential ZIKV symptoms using a symptom journal in between visits.

Pregnancies will be followed by ultrasound at study visits in accordance with local clinical standard of care. All clinical ultrasounds will be recorded; at a minimum one ultrasound per trimester will be obtained. Women will be educated about Zika prevention, signs and symptoms and queried for signs and symptoms of ZIKV infection and pregnancy complications at each visit. Women experiencing Zika-like symptoms in between study visits will be instructed to come to the clinic immediately to provide specimens for testing.

Women testing positive for ZIKV will be scheduled for two additional clinic visits within 2 weeks for collection of additional blood, urine, saliva, vaginal fluid samples and a fetal ultrasound, per country and local guidelines. In addition, the MoH and local health jurisdictions will be informed per in-country regulations/requirements. Following local regulatory requirements, participants will be given their test results and counseled as needed to explain test results. Counseling may include information about the limitations in interpretation of the assays and provision of social support, as appropriate.

Infants will be assessed for ZIKV infection at birth with laboratory assessment from cord blood or peripheral blood, urine, and saliva. They will be evaluated for signs and symptoms of neurological abnormalities and will undergo auditory and visual screening. In addition, infants will be seen at 3, 6, and 12 months for a general physical exam; neurological assessment; auditory, visual, and neurodevelopmental screening assessments; and laboratory testing. If auditory, visual, or neurodevelopmental evaluations are abnormal, infants will be referred for additional testing and results recorded. Data from any radiological testing for infants with ZIKV exposure done for clinical care will be recorded. Infants noted to have any deficits will undergo appropriate follow-up and testing. If cerebrospinal fluid (CSF) is collected per clinical guidelines, remaining CSF will be stored in the study bio-repository for future testing.

See Tables 1 and 2 for additional information about data and specimen collection.

**Table 1:** Data collection details

Assessment	Time frame	Data collected
Maternal Data		
Demographic Survey	Study entry	Basic demographics, e.g., age, race and ethnicity, marital status, and education
Medical History	Study entry	History of: significant medical disorders; previous DENV or Yellow Fever infections and vaccination; any prior infections with parvovirus, herpes, cytomegalovirus, and syphilis
Physical examination and Zika-like symptoms questionnaire	All visits	Assessment of general appearance, including height and weight, and blood pressure and temperature; history of recent symptoms consistent with ZIKV infections, including rash, fever, arthralgias, eye pain, and conjunctivitis
Pregnancy questionnaire	Monthly visits	Signs/ symptoms of pregnancy complications, including decrease in fetal movement, rupture of membranes, vaginal bleeding, headaches, scotomata, nausea, and vomiting.
Imaging assessments	Once per trimester	Ultrasound results
Environmental/ occupational exposure survey	Once per trimester	Participant’s and her domestic partner’s occupational history; household characteristics; behavioral risk factors (e.g. drinking, smoking); exposure to animals in the home; and exposure to known environmental chemicals
Delivery (live birth or fetal loss) information	At delivery	Mode, location, and outcome of the delivery
Infant Data		
Physical examination and growth parameters	All visits	Examination of cardiac, respiratory, and other systems, and skin and gastrointestinal conditions; weight, length or height, and head circumference; gestational age at birth and Apgar scores (birth visit only)
Hearing, ophthalmologic, and neurological assessments	All visits	General motor development markers, including lethargy, seizures, reflexes, etc.; hearing assessments (otoacoustic emission or automated auditory brainstem response); general eye exams; and ophthalmologic exams to detect chorioretinitis or abnormalities in conjunctiva, lens, or retina. If initial exams are abnormal, infants will be referred for additional audiology and/or ophthalmology testing, and results (if available) from any tests done for clinical reasons will be recorded.
Imaging studies	As done per local standard of care	Any imaging assessments done for clinical care, including any neuroimaging studies (ultrasound, CT, or MRI) obtained for clinical reasons to detect neurologic abnormalities such as intracranial calcifications.
Neurodevelopmental assessment	3, 6, and 12- month visits	Screening neurodevelopmental assessments (Ages and Stages Questionnaire or the Bayley Infant Neurodevelopmental Screener) of all study participants. If results of screening assessment are abnormal, the infant will be referred for diagnostic neurodevelopmental testing using the Bayley Scales of Infant and Toddler Development (BSID) -III.

**Table 2:** Specimen collection schedules

Maternal specimen collection schedule							
		First trimester < 14 weeks	Second Trimester < 28 weeks gestation	Third Trimester 28 weeks and greater	Delivery: live birth or fetal loss	6 weeks postpartum +/− 14 days	Zika symptomatic visits
	Screening & Entry	Monthly visits	Monthly visits	Monthly visits			
Serum, plasma, whole blood, urine, saliva, vaginal swab	X	X	X	X	X	X	X
Breast milk					X	X	
Amniotic fluid (if possible)			Only if amniocentesis was performed for clinical care		X		
Cord blood (if possible)					X		
Placenta and fetal tissue if fetal loss (if possible)					X		
Infant specimen collection schedule							
	Birth	3 months		6 months			12 months
Urine, saliva, serum	X	X		X			X
Cerebrospinal fluid (remnant specimen)	Only if collected for clinical care	Only if collected for clinical care		Only if collected for clinical care			Only if collected for clinical care

### Laboratory evaluations, assays, and handling

Maternal participants will be tested for Anti-ZIKV IgM antibodies using a serological assay with Emergency Use Authorization (EUA) approval from the United States Food and Drug Administration (FDA). As more specific/sensitive assays are developed, those assays may be substituted. DENV serology may be run in parallel to the Zika serology. If a routine monthly visit blood sample tests positive (ZIKV IgM and/or other serological assay), an EUA-approved ZIKV molecular assay will be performed for the detection of ZIKV RNA. Timely testing and reporting are a priority but may be limited due to laboratory capacities. All sites will use the same molecular and serological test kits and a standardized protocol.

Among maternal participants, assays to detect antibody to toxoplasma, rubella, CMV, or HSV will be run per local clinical laboratory procedures. The rapid plasma reagin (RPR) test for syphilis will be performed on plasma in accordance with local clinical laboratory procedures. Because false positives may occur, additional testing may be conducted as part of the clinical care. Assays for the detection of antibody (ELISA) or viral RNA (by RT-PCR) may be performed for DENV and in some cases for chikungunya virus (CHIKV) or WNV as appropriate per local epidemiologic disease surveillance report guidelines. Additionally, urine will be collected from maternal participants, at selected sites, to test for environmental contaminants such as phthalates, pesticides, and other chemicals.

Infant birth visit blood specimens will be evaluated by antibody assay for toxoplasma, rubella, CMV, or HSV, per local clinical laboratory procedures. The rapid plasma reagin (RPR) test will be performed on plasma in accordance with local clinical laboratory procedures. Infants will undergo diagnostic testing for ZIKV if they have clinical indication for ZIKV testing (i.e., birth defects consistent with congenital Zika syndrome or they were born to mother with laboratory evidence of possible ZIKV infection during pregnancy).

All biological samples will be received, processed, aliquoted, and stored at each site’s central laboratory. Samples may be shipped from each clinical collection site to the designated central repository for each site in each country following designated study sample shipping procedures.

### Statistical considerations

#### Study outcome measures

The primary composite endpoint is defined as the occurrence of congenital malformations and adverse fetal and neonatal outcomes. This includes microcephaly, fetal demise, neonatal death (within first 28 days of life), CNS malformations, hydrops, ocular and hearing abnormalities, infant seizures and arthrogryposis.

#### Sample size considerations

Recruitment will target enrolling 10,000 pregnant women, in their first or early second trimester of gestation, or women with confirmed acute ZIKV infection at any gestational age, across ten sites. If either the enrollment or infection rate is significantly different from what has been projected, consideration will be given to reducing the sample size, expanding the study to include a larger sample size, adding an additional site, or focusing recruitment on ZIKV-symptomatic women or women in an area where the current incidence rate is high.

#### Sample size calculations for primary objectives

The power of the study to detect a difference was estimated by simulation, using the prevalence of ZIKV infection and adverse fetal outcome (Table 3 and Table 4). These power simulations are based on Fisher’s Exact Tests comparing the rate of adverse outcome among ZIKV infected and uninfected participants, and do not adjust for maternal covariates or differences in prevalence or outcomes among different sites or different countries, as the final analysis will do. All simulations were based on 10,000 repetitions and a two-sided unadjusted alpha level of 0.05.

**Table 3:** Sample size to detect prevalence of primary outcomes among ZIKV infected and uninfected study participants

Proportion of pregnant women with ZIKV infection	Prevalence of congenital malformations and adverse fetal outcomes among ZIKV uninfected women	Prevalence of congenital malformations and adverse fetal outcomes among ZIKV infected women	Total number of women	Power
15%	1%	2%	6000	65%
			8000	77%
			10,000	85%
		3%	6000	98%
	2%	4%	6000	91%
20%	1%	2%	6000	74%
			8000	84%
			10,000	91%
25%			6000	80%
			8000	90%
			10,000	95%
	2%	4%	6000	97%

**Table 4:** Sample size to detect prevalence of primary outcomes among ZIKV infected symptomatic and asymptomatic participants

Proportion of women with ZIKV symptoms	Prevalence of congenital malformations and adverse fetal outcomes among uninfected and asymptomatic women	Prevalence of congenital malformations and adverse fetal outcomes among ZIKV symptomatic women	Total number of women	Power
3%	1%	3%	6000	57%
			8000	69%
			10,000	77%
4%			6000	68%
			8000	78%
			10,000	85%
5%			6000	75%
			8000	84%
			10,000	91%
		4%	6000	94%
			8000	97%
			10,000	99%

Table 3 shows the sample size needed to detect a difference in the prevalence of ZIKV exposure in fetuses and infants of women who become infected with ZIKV compared to women who do not with 90% power, under a range of assumptions. For example, with a 25% prevalence rate of ZIKV infection among pregnant women, a 1% rate of the composite fetal outcome among uninfected women and a 2% rate of this outcome among women with ZIKV infection, we would need at least 8000 women to have 90% power to detect a difference. Assuming a 25% lost to follow-up rate in our sample, this suggests enrolling at least 10,000 women.

Table 4 shows the power of the study to detect a difference in the incidence of congenital malformations and adverse fetal outcomes in fetuses and infants of women who become symptomatic with ZIKV infection compared to women who do not, under a range of assumptions.

A key secondary objective is the association between gestational age at time of ZIKV infection and risk of adverse fetal outcome. With 10,000 women enrolled, if we assume that ZIKV exposure will be approximately uniformly distributed across trimesters and 25% of women become ZIKV positive, we would expect 800–900 women to be infected during their first trimester. If the rate of the composite endpoint among these first-trimester infections was 3%, compared to 1% in those without ZIKV infection, we would have high (> 95%) power to detect a difference. We would also have reasonable power to detect a difference between these first trimester infections and a similar number of infections at later trimesters if the increased risk is limited to the first trimester, if the trimester of ZIKV infection can be confirmed in a large enough group of ZIKV-infected women.

### Analyses

The primary analyses will use an indicator of the primary composite endpoint as the response and an indicator of either ZIKV infection with or without ZIKV symptoms as the predictor of interest. These models will be adjusted for site, maternal age at enrollment, season or month of enrollment, and documented infections with CMV and DENV. Additional covariates may be included but will be pre-specified. Participants that are lost to follow-up, outcomes that are missing because of fetal loss before 20 weeks, and propensity for exposure will be adjusted for appropriately. Sensitivity analyses will include alternate models that examine the assumptions behind the primary analysis. A key secondary analysis is the effect of trimester of infection on probability of outcome. This association will be estimated using a similar model to the primary analysis but with an additional indicator variable for trimester of infection. Another key analysis is the comparison of rates of fetal loss between women who experience ZIKV infection with symptoms and those who do not. This will also be fit using similar models to the primary analysis, accounting for loss to follow-up and probability of exposure before 20 weeks. Additional analyses will be carried out to compare the rates in secondary/exploratory outcomes between those ZIKV-infected versus uninfected mothers or between those with and without ZIKV symptoms.

### Ethical standard

The investigators will ensure that this study is conducted in full conformity with the principles set forth in The Belmont Report: Ethical Principles and Guidelines for the Protection of Human Subjects of Research of the U.S. National Commission for the Protection of Human Subjects of Biomedical and Behavioral Research (April 18, 1979) and codified in 45 CFR Part 46 or the International Conference on Harmonization (ICH) E6; 62 Federal Regulations 25691 (1997). The participating institutions will hold a current Federal-Wide Assurance (FWA) issued by Office for Human Research Protections for U.S. government–funded research. Prior to enrollment of participants, the approved protocol, associated informed consent documents, participant counseling scripts, recruitment material, and surveys for participants will be reviewed and approved by the appropriate IRB/IEC listed on the institution’s FWA.

### Data management

RTI International (RTI) will be responsible for data management processes and serve as the central data coordinating center (DCC). RTI will develop a data collection and management system to enhance the uniformity of data and facilitate cross-site analyses. Research Electronic Data Capture (REDCap) will serve as the core data collection component of the data management system [33]. Recognizing the site-specific logistical requirements, the system will be integrated with the existing site systems and processes as much as possible to maximize the utility of available infrastructure and experience. Based on site-specific needs, data may be captured on handheld devices (e.g., tablets) or paper and entered into the data management system at each site. Laboratory assay results and specimen storage data will be imported into the REDCap database. The DCC will provide standardized progress reports to the study sponsors and the site investigators monthly to monitor the quality of data collected, including completion of key variables and adverse events. The site record maintenance will be compliant with ICH E6 section 4.9 and regulatory and institutional requirements for the protection of participant confidentiality.

## Discussion

With the recent emergence of ZIKV in the Americas and its association with adverse pregnancy outcomes in this region, a much better understanding of the range of clinical outcomes associated with exposure to ZIKV during pregnancy is needed. Unanswered questions include the impact of timing of the exposure, the potential ZIKV interaction with other factors including environmental exposures, other infections, or socio-demographic factors, and the spectrum of adverse birth and infant outcomes. The ZIP study, with the unique opportunity to prospectively follow 10,000 pregnant women residing in ZIKV regions in Latin America and the Caribbean, has evolved with the changing distribution of ZIKV infection throughout the region and will provide important data to inform our understanding of ZIKV and pregnancy outcomes. It will also provide data to characterize the range of clinical sequelae that may occur among ZIKV-exposed infants. This study will serve as a platform for future research to determine the most effective practices and technologies for the diagnosis of ZIKV infection and evaluation of ZIKV-related outcomes in exposed mothers and their children.

## Data Availability

The protocol (version 1.0, February 1, 2017) for this study is listed on clinicaltrials.gov (ClinicalTrials.gov Identifier: NCT02856984). De-identified data from this study will be made publicly available on the NICHD Data and Specimen Hub (DASH) platform in early 2020.

## Abbreviations

CDC: Centers for Disease Control and Prevention
CFR: Code of Federal Regulations
CKNV: Chikungunya virus
CMV: Cytomegalovirus
CNS: Central nervous system
CRF: Case Report Form
CSF: Cerebrospinal fluid
DCC: Data Coordinating Center
DENV: Dengue virus
DMS: Data management system
ELISA: Enzyme linked immunosorbent assay
EUA: Emergency Use Authorization
FDA: Food and Drug Administration
FIOCRUZ: Fundação Oswaldo Cruz
FWA: Federal-Wide Assurance
GCP: Good Clinical Practice
HSV: Herpes simplex virus
ICH: International Conference on Harmonization
IEC: Independent or Institutional Ethics Committee
IRB: Institutional Review Board
MoH: Ministry of Health
NIAID: National Institute of Allergy and Infectious Diseases
NICHD: National Institute of Child Health and Human Development
NIH: National Institutes of Health
PCR: Polymerase chain reaction
REDCap: Research Electronic Data Capture
RPR: Rapid plasma reagin
RT-PCR: Reverse transcription polymerase chain reaction
RUDC: Research Unit Data Center
SOP: Standard Operating Procedure
WHO: World Health Organization
WNV: West Nile virus
ZIKV: Zika virus

## References

[1] Hurtado-Villa Paula, Puerto Angie K., Victoria Salomé, Gracia Gloria, Guasmayán Lesly, Arce Patricia, Álvarez Gilberto, Blandón Esperanza, Rengifo Nubia, Holguín Jorge A., Durán Alexander, Zarante Ignacio (2017). Raised Frequency of Microcephaly Related to Zika Virus Infection in Two Birth Defects Surveillance Systems in Bogotá and Cali, Colombia. The Pediatric Infectious Disease Journal.

[2] Moura da Silva AA, Ganz JS, Sousa PD, Doriqui MJ, Ribeiro MR, Branco MD (2016). Early Growth and Neurologic Outcomes of Infants with Probable Congenital Zika Virus Syndrome. Emerg Infect Dis.

[3] Petersen LR (2016). Zika Virus. N Engl J Med.

[4] Paixao ES (2016). History, epidemiology, and clinical manifestations of Zika: a systematic review. Am J Public Health.

[5] Fauci AS, Morens DM (2016). Zika virus in the Americas--yet another arbovirus threat. N Engl J Med.

[6] Hayes EB (2009). Zika virus outside Africa. Emerg Infect Dis.

[7] Weaver SC (2016). Zika virus: history, emergence, biology, and prospects for control. Antivir Res.

[8] Mallet HP, Vial AL, Musso D (2016). Bilan de l’épidémie à virus Zika survenue en Polynésie française entre octobre 2013 et mars 2014. De la description de l’épidémie aux connaissances acquises après l’évènement. Bull Epidemiol Hebd.

[9] Duffy MR (2009). Zika virus outbreak on Yap Island, Federated States of Micronesia. N Engl J Med.

[10] Cao-Lormeau V-M (2016). Guillain-Barré Syndrome outbreak associated with Zika virus infection in French Polynesia: a case-control study. Lancet.

[11] Brasil P (2016). Zika virus infection in pregnant women in Rio de Janeiro. N Engl J Med.

[12] Kleber de Oliveira W (2016). Increase in reported prevalence of microcephaly in infants born to women living in areas with confirmed Zika virus transmission during the first trimester of pregnancy - Brazil, 2015. MMWR Morb Mortal Wkly Rep.

[13] de Araújo TVB (2018). Association between microcephaly, Zika virus infection, and other risk factors in Brazil: final report of a case-control study. Lancet Infect Dis.

[14] Rasmussen SA (2016). Zika virus and birth defects — reviewing the evidence for causality. N Engl J Med.

[15] Besnard M, Eyrolle-Guignot D, Guillemette-Artur P, Lastère S, Bost-Bezeaud F, Marcelis L, Abadie V, Garel C, Moutard M, Jouannic J, Rozenberg F, Leparc-Goffart I, Mallet H. Congenital cerebral malformations and dysfunction in fetuses and newborns following the 2013 to 2014 Zika virus epidemic in French Polynesia. Euro Surveill. 2016;21(13). 10.2807/1560-7917.ES.2016.21.13.30181. 10.2807/1560-7917.ES.2016.21.13.30181 27063794

[16] Honein MA (2017). Birth defects among fetuses and infants of US women with evidence of possible Zika virus infection during pregnancy. Jama.

[17] Cuevas EL (2016). Preliminary report of microcephaly potentially associated with Zika virus infection during pregnancy - Colombia, January-November 2016. MMWR Morb Mortal Wkly Rep.

[18] Hoen B (2018). Pregnancy outcomes after ZIKV infection in French territories in the Americas. N Engl J Med.

[19] Reynolds MR (2017). Vital signs: update on Zika virus-associated birth defects and evaluation of all U.S. infants with congenital Zika virus exposure - U.S. Zika pregnancy registry, 2016. MMWR Morb Mortal Wkly Rep.

[20] Costa F (2016). Emergence of congenital Zika syndrome: viewpoint from the front lines. Ann Intern Med.

[21] Moore CA (2017). Characterizing the pattern of anomalies in congenital zika syndrome for pediatric clinicians. JAMA Pediatr.

[22] Del Campo M (2017). The phenotypic spectrum of congenital Zika syndrome. Am J Med Genet A.

[23] Microcephaly Epidemic Research Group (2016). Microcephaly in infants, Pernambuco state, Brazil, 2015. Emerg Infect Dis J.

[24] de Paula Freitas Bruno, de Oliveira Dias João Rafael, Prazeres Juliana, Sacramento Gielson Almeida, Ko Albert Icksang, Maia Maurício, Belfort Rubens (2016). Ocular Findings in Infants With Microcephaly Associated With Presumed Zika Virus Congenital Infection in Salvador, Brazil. JAMA Ophthalmology.

[25] Zin AA (2017). Screening criteria for ophthalmic manifestations of congenital Zika virus infection. JAMA Pediatr.

[26] Leal MC (2016). Hearing loss in infants with microcephaly and evidence of congenital Zika virus infection - Brazil, November 2015-may 2016. MMWR Morb Mortal Wkly Rep.

[27] Guillemette-Artur P (2016). Prenatal brain MRI of fetuses with Zika virus infection. Pediatr Radiol.

[28] de Fatima Vasco Aragao M (2016). Clinical features and neuroimaging (CT and MRI) findings in presumed Zika virus related congenital infection and microcephaly: retrospective case series study. BMJ.

[29] van der Linden V (2016). Description of 13 infants born during October 2015-January 2016 with congenital Zika virus infection without microcephaly at birth - Brazil. MMWR Morb Mortal Wkly Rep.

[30] Cerbino-Neto J (2016). Clinical manifestations of Zika virus infection, Rio de Janeiro, Brazil, 2015. Emerg Infect Dis.

[31] Sarno M (2016). Zika virus infection and stillbirths: a case of Hydrops Fetalis, Hydranencephaly and fetal demise. PLoS Negl Trop Dis.

[32] Oduyebo T (2016). Update: interim guidelines for health care providers caring for pregnant women and women of reproductive age with possible Zika virus exposure - United States, 2016. MMWR Morb Mortal Wkly Rep.

[33] Harris PA (2009). Research electronic data capture (REDCap)—a metadata-driven methodology and workflow process for providing translational research informatics support. J Biomed Inform.

