# Correction to: International prospective observational cohort study of Zika in infants and pregnancy (ZIP study): study protocol

**DOI:** 10.1186/s12884-019-2589-8

**Published:** 2019-11-19

**Authors:** Jill F. Lebov, Juan F. Arias, Angel Balmaseda, William Britt, José F. Cordero, Luiz Augusto Galvão, Ana Lucía Garces, K. Michael Hambidge, Eva Harris, Albert Ko, Nancy Krebs, Ernesto T. A. Marques, Alexander M. Martinez, Elizabeth McClure, Democrito B. Miranda-Filho, Maria Elisabeth Lopes Moreira, Marisa M. Mussi-Pinhata, Theresa J. Ochoa, Jorge E. Osorio, Deolinda M. F. Scalabrin, Stacey Schultz-Cherry, George R. Seage, Kristen Stolka, César Augusto Ugarte-Gil, Carmen Milagros Velez Vega, Michael Welton, Ricardo Ximenes, Carmen Zorrilla

**Affiliations:** 1 Social, Statistical and Environmental Sciences, RTI International, Durham, NC USA; 2 Department of Infectious Diseases, St Jude Children’s Research Hospital, Memphis, TN 38105 USA; 3 Centro Nacional de Diagnostico y Referencia, Complejo Nacional de Salud, Managua, Nicaragua; 4 Department of Pediatrics, University of Alabama at Birmingham, Birmingham, AL USA; 5 Department of Epidemiology and Biostatistics, College of Public Health, University of Georgia, Athens, GA USA; 6 Center for Global Health – CRIS, FIOCRUZ, Rio de Janeiro, Brazil; 7 Fundación para la Alimentación y Nutrición de Centro América y Panamá (INCAP), Guatemala City, Guatemala; 8 Section of Nutrition, Pediatrics, University of Colorado, Aurora, CO USA; 9 Division of Infectious Diseases and Vaccinology, School of Public Health, University of California, Berkeley, CA USA; 10 Department of Epidemiology of Microbial Diseases, Yale School of Public Health, New Haven, CT USA; 11 Instituto Gonçalo Moniz, Fundação Oswaldo Cruz/MS, Salvador, Brazil; 12 School of Public Health, University of Pittsburgh, Pittsburgh, PA USA; 13 Instituto Aggeu Magalhães, Department of Virology and Experimental Therapeutics, FIOCRUZ, Pernambuco, Brazil; 14 Director of Research Institute at Imbanaco Medical Center, Cali, Colombia; 15 Programa de Pós-Graduação em Ciências da Saúde (PPGCS) da Universidade de Pernambuco, Microcephaly Epidemic Research Group, Recife, Brazil; 16 Instituto Fernandes Figueira – FIOCRUZ, Rio de Janeiro, Brazil; 17 Ribeirão Preto Medical School, Ribeirão Preto, Brazil; 18 Instituto de Medicina Tropical Alexander von Humboldt and Facultad de Medicina, Universidad Peruana Cayetano Heredia, Lima, Peru; 19 Department of Pathobiological Sciences, University of Wisconsin, Madison, WI USA; 20 Department of Epidemiology, Harvard Chan School of Public Health, Boston, MA USA; 21 University of Puerto Rico, San Juan, Puerto Rico; 22 Departamento de Medicina Tropical da Universidade Federal de Pernambuco, Microcephaly Epidemic Research Group, Recife, Brazil; 23 Maternal-Infant Studies Center (CEMI), San Juan, Puerto Rico

**Correction to: BMC Pregnancy Childbirth**

**https://doi.org/10.1186/s12884-019-2430-4**

Following publication of the original article [1], the author mentioned that two additional NIH staff were involved in the development of the protocol who did not receive recognition in the Acknowledgments section in their published article. He would like to add the following people to the **Acknowledgements section**:

- **Martha Nason** and **Erin Gabriel**

The publisher apologizes for any inconvenience caused by this error.
